# The role of putrescine in the regulation of proteins and fatty acids of thylakoid membranes under salt stress

**DOI:** 10.1038/srep14390

**Published:** 2015-10-05

**Authors:** Sheng Shu, Yinghui Yuan, Jie Chen, Jin Sun, Wenhua Zhang, Yuanyuan Tang, Min Zhong, Shirong Guo

**Affiliations:** 1 Key Laboratory of Southern Vegetable Crop Genetic Improvement in Ministry of Agriculture, College of Horticulture, Nanjing Agricultural University, Nanjing 210095, People’s Republic of China; 2 College of Life Sciences, Nanjing Agricultural University, Nanjing 210095, People’s Republic of China

## Abstract

Polyamines can alleviate the inhibitory effects of salinity on plant growth by regulating photosynthetic efficiency. However, little information is available to explain the specific mechanisms underlying the contribution of polyamines to salt tolerance of the photosynthetic apparatus. Here, we investigated the role of putrescine (Put) on the photosynthetic apparatus of cucumber seedlings under salt stress. We found that NaCl stress resulted in severe ion toxicity and oxidative stress in cucumber chloroplasts. In addition, salinity caused a significant increase in the saturated fatty acid contents of thylakoid membranes. Put altered unsaturated fatty acid content, thereby alleviating the disintegration of thylakoid grana lamellae and reducing the number of plastoglobuli in thylakoid membranes. BN-PAGE revealed Put up-regulated the expression of ATP synthase, CP47, D1, Qb, and psbA proteins and down-regulated CP24, D2, and LHCII type III in NaCl-stressed thylakoid membranes. qRT-PCR analysis of gene expression was used to compare transcript and protein accumulation among 10 candidate proteins. For five of these proteins, induced transcript accumulation was consistent with the pattern of induced protein accumulation. Our results suggest that Put regulates protein expression at transcriptional and translational levels by increasing endogenous polyamines levels in thylakoid membranes, which may stabilise photosynthetic apparatus under salt stress.

Salt stress is one of the most serious environmental factors limiting agricultural crop productivity. Reduced plant growth due to salt stress often result in significant inhibition of their photosynthetic activity^1^. Salt-induced inhibition of photosynthetic activity may result from closure of stomata induced by osmotic stress^2^, reduced efficiency of ribulose-1,5-bisphosphate carboxylase/oxygenase (RuBisco) for carbon assimilation^3^, and disruption of photosynthetic systems, chloroplast structure/function or thylakoid membrane organization by excessive energy^1^,^2^,^4^. Many attempts have been made to improve the photosynthetic capacity of a variety of crops or cultivars under salinity conditions^1^,^5^. These efforts include traditional breeding programs, transgenic approaches, and exogenous polyamine application.

Polyamines (PAs) are acknowledged regulators of plant growth, development, and stress responses. The most common PAs in higher plants are triamine spermidine (Spd), tetraamine spermine (Spm), and their diamine obligate precursor putrescine (Put)^6^. Hamdani *et al.*^7^ reported that these three major PAs are normally found in the photosynthetic apparatus of higher plants and are implicated in plant growth and stress responses. Several lines of evidence support the relationship between PAs and photosynthesis under stress conditions. It has been suggested that the exogenous application of PAs can, to some extent, alleviate salinity-induced decreases in photosynthetic efficiency of higher plants, but this effect strongly depends on both PA concentrations or types, and stress levels^8^,^9^. It has been reported that PAs prevent chlorophyll loss from thylakoid membranes by stabilizing photosystem complexes during senescence^10^. Exogenous application of Spd has been shown to alleviate the degradation of D1, D2, and cytochrome *f* as well as the transcripts of their corresponding genes in oat leaves under drought and osmotic stresses^11^,^12^. Sen *et al.*^13^ found that addition of Spd to the alga of the lichen *Xanthoria parietina* resulted in an increase in the transcript level of D1 in photosystem II (PSII). The protective action of Spd in PSII can be explained by the involvement of PAs in the modulation of transcription and translation of these proteins.

Because of their polycationic nature at physiological pH, PAs are able to interact with negatively charged macromolecules such as proteins, nucleic acids, and chromatin, thereby stabilizing their structures. The role of PAs in stress resistance and their association with thylakoid membrane proteins indicate that PAs are also likely to interact directly with photosystem components. Yaakoubi *et al.*^14^ demonstrated that Spm can interact with photosystem I (PSI) sub-membrane fractions and protect PSI photochemical activity against photoinhibition induced by high light stress. Application of exogenous Put has been reported to alter the content of light-harvesting complex II (LHCII) monomers and oligomers as well as PSI and PSII core proteins in thylakoid membranes of *Scenedesmus*, thereby improving photo-adaptability of that species^15^. The response of the photosynthetic apparatus to stress conditions is affected by changes in the expression of LHCII-associated Put and Spm, which have been shown to adjust the size of the LHCII^16^,^17^. Shu *et al.*^1^ have shown that Put increases endogenous PA levels in chloroplasts and can help overcome the damaging effects of salt stress on photochemical efficiency. The relationship between increased PA levels in thylakoids and LHCII antenna size suggests that thylakoid-bound PAs exert a regulatory role in the adaptation process of the photosynthetic apparatus^1^,^7^.

Although the potential roles of PAs in the protection of photosynthetic apparatus responses to abiotic stresses have been well studied, few reports have focussed on the specific mechanism underpinning the action of PAs on thylakoid membrane proteins of the photosynthetic apparatus of higher plants exposed to salt stress. Put, can be converted to Spd, and then to Spm by the successive addition of aminopropyl groups from decarboxylated S-adenosylmethionine. Cucumber (*Cucumis sativus*) is an economically important horticultural crop that is highly sensitive to salinity, especially at the seedling stage. Our previous results indicated that electrostatic interactions of PAs or their conjugation with proteins of PSII may help stabilize protein conformational structure and enhance chloroplast function in salt-stressed cucumber seedlings^1^,^8^. In the present study, the effects of Put on the accumulation of toxic ions (Na^+^ and Cl^−^) and reactive oxygen species (ROS) (mainly H_2_O_2_) were analyzed in control and NaCl-treated cucumber chloroplasts. The regulatory interaction of Put with protein and fatty acid components of the thylakoid membranes was also considered. The results of this study provide insights into the mechanism by which PAs protect the structure and functions of the photosynthetic apparatus from NaCl-induced damage.

## Results

### Na^+^ and Cl^**−**^ concentrations in chloroplasts

**Figure 1:**
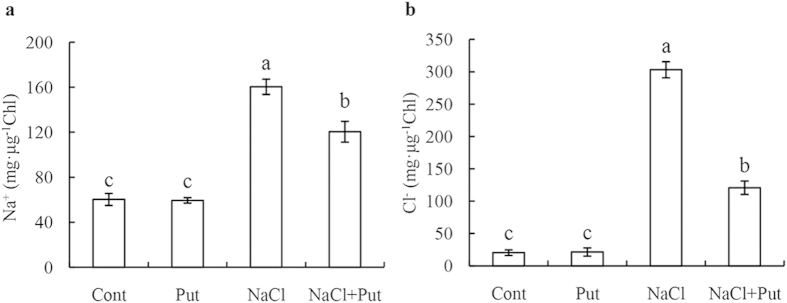
Effects of exogenous putrescine (Put) on Na^+^ **(a)** and Cl^−^ **(b)** concentrations in the chloroplasts of cucumber plants grown in nutrient solutions with or without NaCl for 7 days. Each histogram represents a mean ± SE of three independent experiments. Different letters indicate significant differences between treatments (*P* < 0.05). The data were taken on the third leaves (numbered basipetally) after the final salt treatment (75 mM NaCl). Cont, 0 mM NaCl + 0 mM Put; Put, 0 mM NaCl + 8 mM Put; NaCl, 75 mM NaCl + 0 mM Put; NaCl+Put, 75 mM NaCl + 8 mM Put.

Na^+^ and Cl^−^ concentrations in cucumber chloroplasts were measured on day 7 after the final salt treatment (75 mM NaCl) with or without Put. We found that there were significant increases in the contents of Na^+^ (Fig. 1a) and Cl^−^ (Fig. 1b) of the NaCl-stressed chloroplasts compared with the controls (1386.7% and 166.2% respectively). However, application of 8 mM Put dramatically decreased the levels of accumulated Na^+^ and Cl^−^ in the salt-stressed chloroplasts. No significant differences were observed in the Na^+^ and Cl^−^ concentrations of the chloroplasts between the control and the control plus Put treatment.

### H_2_O_2_ levels in guard cell chloroplasts

**Figure 2:**
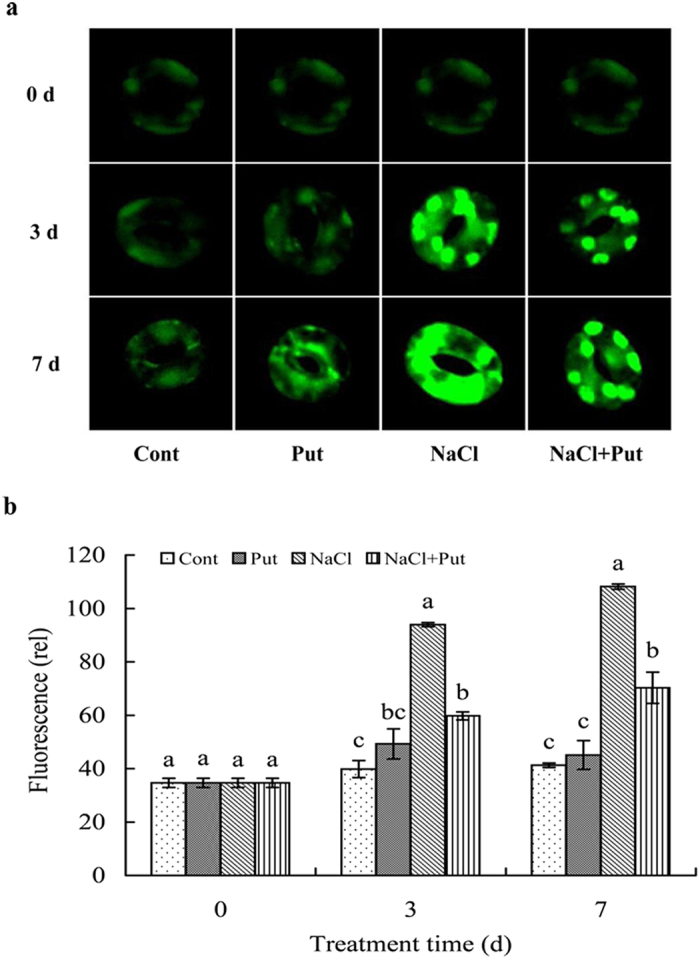
(**a**) Effects of exogenous putrescine (Put) on ROS levels in the chloroplasts of cucumber plants grown in nutrient solutions with or without NaCl for 0, 3 and 7 days. The lower epidermis of the third leaves were torn off and incubated with 2′, 7′-dichlorofluorescein diacetate (DCF-DA). Changes of fluorescence intensity in stomatal cells were observed using a Leica TCS-SP confocal laser scanning microscope. (**b**) Intensity of DCF-DA fluorescence was analysed using Image Leica SP2 software and was expressed as relative value. Each histogram represents a mean ± SE of three independent experiments. Different letters indicate significant differences between treatments (*P* < 0.05).

H_2_O_2_ levels in lower epidermal guard cells were monitored as the fluorescent signals of specific fluorescent probes, DCF-DA (Fig. 2a). DCF-DA-derived H_2_O_2_ signals were quantified as shown in Fig. 2b. We found that there was a marked increase of H_2_O_2_ production in NaCl-stressed chloroplasts of stomatal cells compared with the controls. When the treatment time was extended, the fluorescence intensity of the salt-stressed plants increased gradually. The fluorescence intensity values were significantly higher than those of the control plants during the first 3 days of NaCl treatment, indicating that NaCl stress caused H_2_O_2_ accumulation within chloroplasts. The increase in H_2_O_2_ accumulation was alleviated by application of Put to NaCl-treated plants. Fluorescence intensity in NaCl-treated plants was significantly weakened by Put treatment on days 3 and 7, implying that Put reduced H_2_O_2_ accumulation. Interestingly, Put sprayed onto non-stressed seedlings appeared to increase H_2_O_2_ levels with the extension of treatment time (Fig. 2a), however, when the fluorescence intensities were quantified, H_2_O_2_ levels between the control and Put-treated plants were not statistically different.

### Fatty acid composition of thylakoid membranes

The fatty acids of thylakoid membrane lipids of cucumber leaves were separated and analyzed by gas chromatography. As shown in Table 1, three saturated fatty acids (tetradecanoic acid, palmitic acid, and stearic acid) and five unsaturated ones (palmitoleic acid, hexadecatrienoic acid, oleic acid, linoleic acid, and linolenic acid) were the main fatty acids among the thylakoid membrane lipids. When exposed to 75 mM NaCl for 7 days, the total fatty acid content of the thylakoid membranes was reduced compared with that of the control plants. NaCl stress significantly increased saturated fatty acid levels, but decreased both the unsaturated fatty acid content and the ratio of unsaturated to saturated fatty acids. Application of Put to NaCl-stressed plants increased total and unsaturated fatty acid levels and reduced saturated fatty acid content. Specifically, in response to NaCl treatment, tetradecanoic and palmitic acid content was increased, whereas stearic acid, palmitoleic acid, hexadecatrienoic acid, oleic acid, linoleic acid, and linolenic acid content was decreased by 33.6%, 71.9%, 58.6%, 61.3%, 58.1%, and 83.5% respectively, compared with control plants. When Put was sprayed on the NaCl-stressed plants, an opposite trend was observed. All the results suggest that Put alleviates salt stress-induced thylakoid membrane lipid peroxidation by enhancing unsaturated fatty acid content.

### Chloroplast ultrastructure

**Figure 3:**
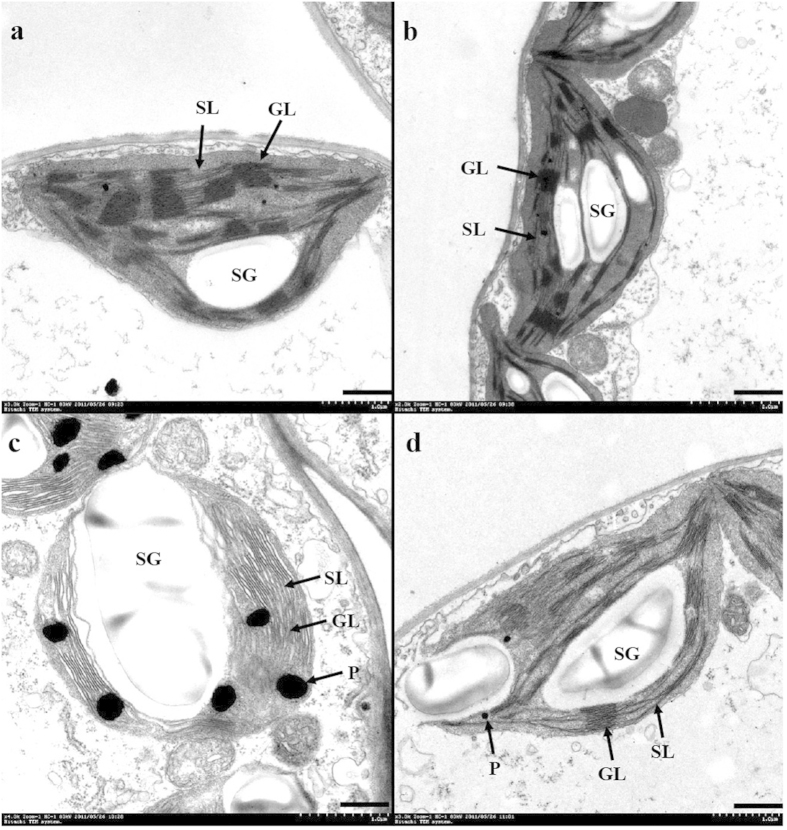
Effects of exogenous putrescine (Put) on the ultrastructure of photosynthetic apparatus in leaves of cucumber plants grown in nutrient solutions with or without NaCl. The third leaves (numbered basipetally) were sampled for ultramicroscopic observation on day 7 after the final concentration salt treatment (75 mM NaCl). SL, stroma lamella; GL, grana lamellae; SG, starch grain; P, plastoglobule. Scale bars for the photosynthetic apparatus are 500 nm. (**a**) Cont (image cited from Shu *et al.*^8^), 0 mM NaCl+0 mM Put; (**b**) Put, 0 mM NaCl+8 mM Put; **(c)** NaCl, 75 mM NaCl + 0 mM Put; (**d**) NaCl+Put, 75 mM NaCl + 8 mM Put.

The ultrastructures of chloroplasts from NaCl and/or Put treated seedlings was investigated using transmission electron microscopy. As shown in Fig. 3, after 7 days of 75 mM NaCl treatment, the ultrastructure of cucumber chloroplasts changed significantly (Fig. 3c). Under salt stress, grana thylakoid lamellae were compressed, chloroplasts were severely deformed into irregular shapes, and many starch granules accumulated. In addition, salt stress induced a separation between cell membranes and chloroplasts, with a large number of osmiophilic particles appearing on the membrane layer of grana and stroma thylakoids. Further observation of the structure of the salt-stressed thylakoid membrane revealed that the grana thylakoid lamellae were disordered and the stroma thylakoid lamellae were fractured. These results suggest that salt stress causes lipid oxidation and structural damage to photosynthetic membranes. Application of exogenous Put alleviated the disintegration of grana thylakoids induced by salt stress (Fig. 3d). Put also decreased the number of osmiophilic particles on grana and stroma thylakoids, thereby maintaining an orderly arrangement between these two structures. In addition, Put restored the connection between chloroplasts and cell membranes in salt-stressed cucumber leaves.

### Blue native(BN)/SDS-PAGE electrophoresis

**Figure 4:**
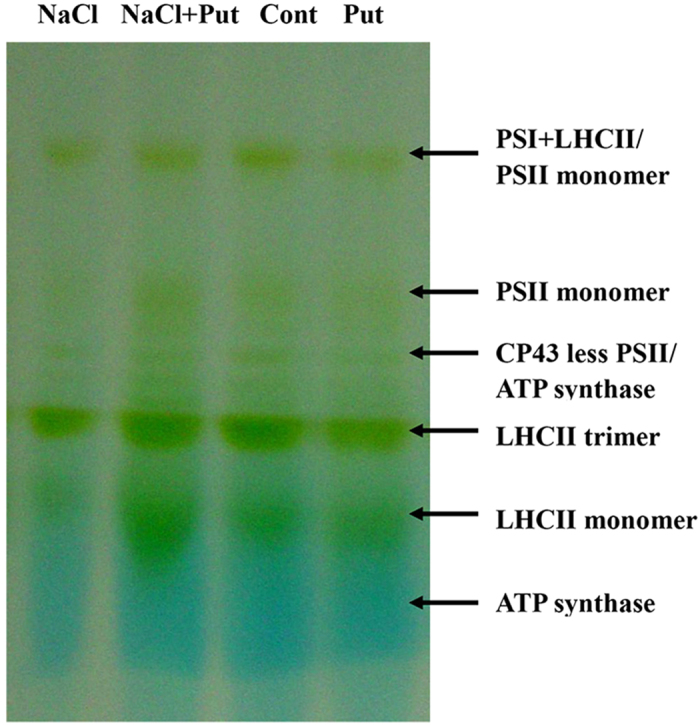
Effect of exogenous putrescine (Put) on thylakoid membrane proteins in cucumber seedlings exposed to 75 mM NaCl for 7 days. Blue-native gel electrophoresis of membrane complexes protein from stroma thylakoids were solubilised by n-dodecylmaltoside. Protein complexes were first solubilized from thylakoid membranes using *n*-dodecyl-β-D-maltoside (DM) and then separated by BN-PAGE. After the first dimensional separation, six major protein complexes were obtained. Mass spectrometric analysis identified the complexes as PSI-LHCII/PSII monomer, PSII, PSI/PSII, ATP synthase, and LHCII trimer. Each lane was loaded with 20 μg Chl for the first dimension. Cont, 0 mM NaCl + 0 mM Put; Put, 0 mM NaCl + 8 mM Put; NaCl, 75 mM NaCl + 0 mM Put; NaCl+Put, 75 mM NaCl + 8 mM Put.

To identify Put-regulated salt stress-mediated thylakoid membrane proteins, we carried out a comparative proteomic analysis of thylakoid membranes after 7 days of treatment. Protein complexes were first solubilized from thylakoid membranes using *n*-dodecyl-β-D-maltoside and then separated by BN-PAGE. After the first dimensional separation, six major protein complexes were obtained (Fig. 4). Mass spectrometric analysis identified the complexes as PSI-LHCII/PSII monomer, PSII, PSI/PSII, ATP synthase, and LHCII trimer. Salinity significantly decreased levels of PSII protein complex and monomeric LHCII bands, but increased trimeric LHCII bands. Application of exogenous Put remarkably increased PSII protein complex and ATP synthase bands and decreased trimeric LHCII bands in the salt-stressed thylakoid membranes.

**Figure 5:**
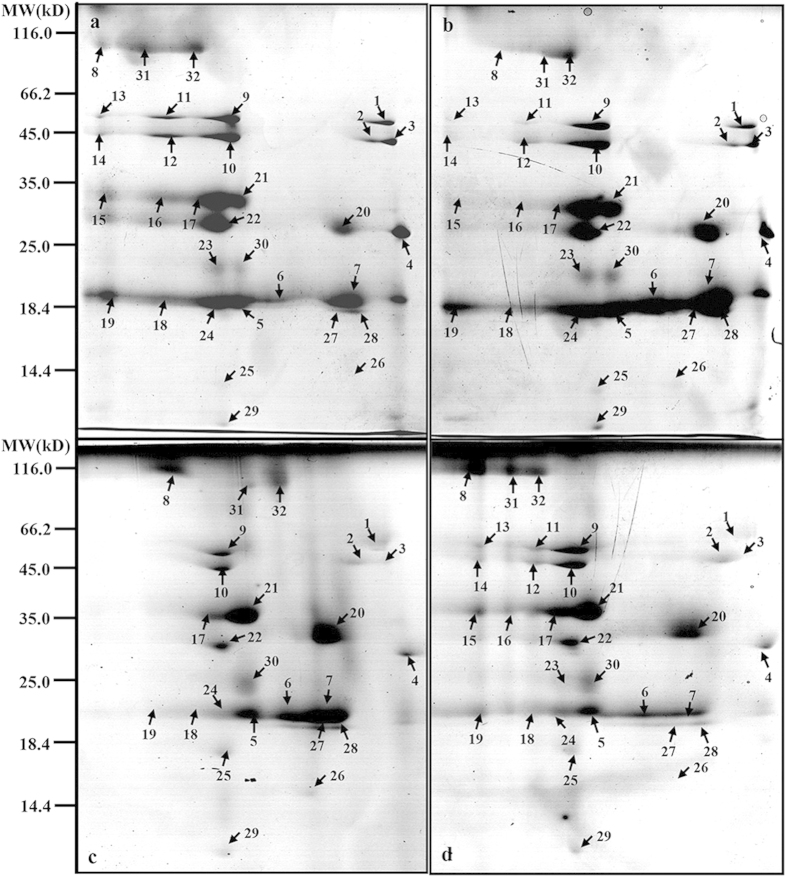
Effect of exogenous putrescine (Put) on BN–SDS-PAGE of thylakoid membrane proteins in leaves of cucumber plants grown in nutrient solutions with or without 75 mM NaCl for 7 days. This separation allowed the subunit patterns of the complexes to be visualised. Analysis of the BN-PAGE gel using Imagemaster 2D Platinum software revealed more than 60 CBB-stained protein spots associated with molecular masses of 14.4–116 kDa. Of these, 32 protein spots differentially regulated in response to NaCl and NaCl+Put treatment were excised from the gels and identified by MALDI-TOF-MS and LC-ESI-MS/MS. Additional information on these identified proteins is summarised in Table 2. (**a**) Cont, 0 mM NaCl + 0 mM Put; (**b**) Put, 0 mM NaCl+8 mM Put; (**c**) NaCl, 75 mM NaCl + 0 mM Put; (**d**) NaCl+Put, 75 mM NaCl + 8 mM Put.

To fingerprint these complexes, BN-PAGE gels were excised and layered onto PAGE gel slabs and then subjected to SDS-urea-PAGE followed by Coomassie blue G-250 staining. This separation enabled visualization of the subunit patterns of the complexes (Fig. 4). Analysis of the BN-PAGE gel using ImageMaster 2D Platinum software revealed more than 60 CBB-stained protein spots associated with molecular masses of 14.4–116 kDa (Fig. 5a–d). Of these, 32 protein spots differentially regulated in response to NaCl and NaCl+Put were excised from the gels and identified by MALDI-TOF-MS and LC-ESI-MS/MS. A MASCOT search against the NCBI database revealed the identity of 29 of the 32 proteins. The remaining three proteins had matching scores significantly lower than the threshold and may represent unknown proteins. Information on all identified protein spots is presented in Table 2.

**Figure 6:**
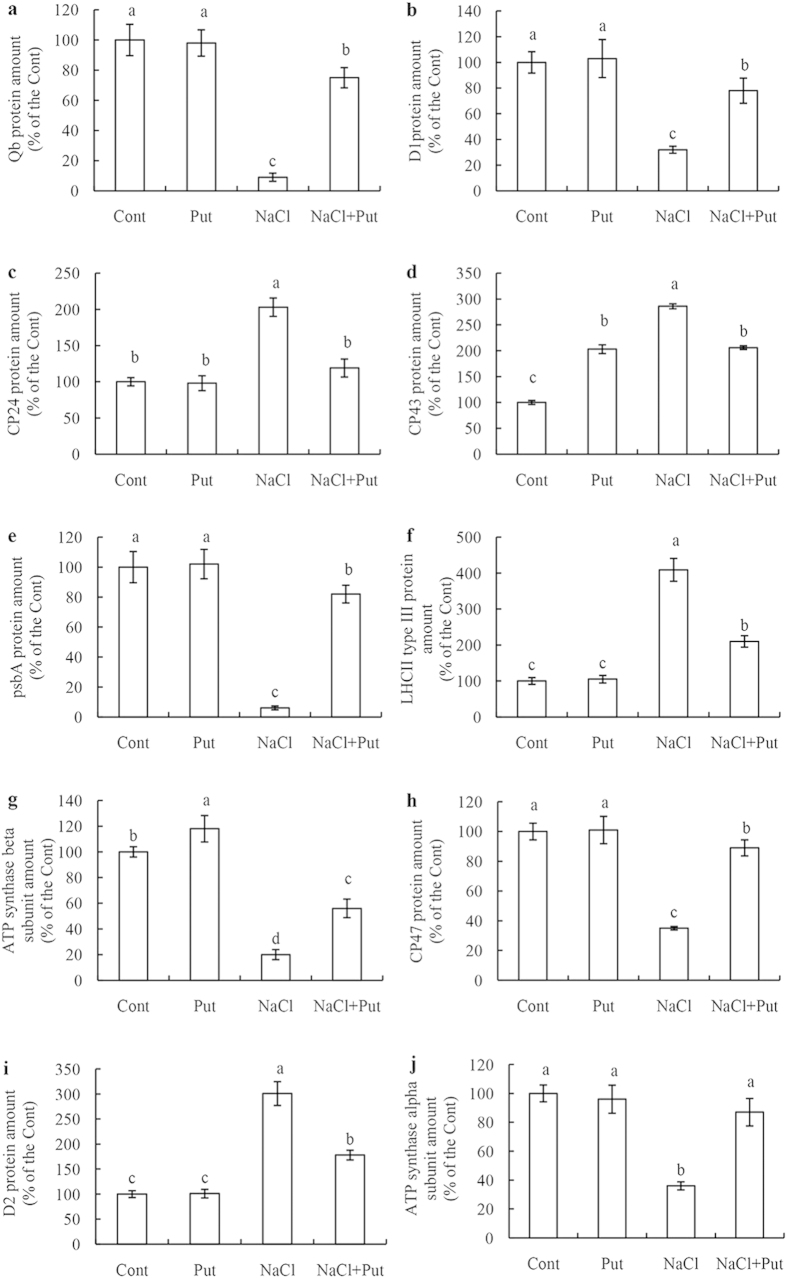
Effect of exogenous putrescine (Put) on abundance of 10 thylakoid membrane proteins in leaves of cucumber plants grown in nutrient solutions with or without NaCl. Each histogram represents a mean ± SE of three independent experiments. Different letters indicate significant differences between treatments (*P* < 0.05). The data were taken on the third leaves (numbered basipetally), after the final salt treatment (75 mM NaCl) for 7 days. Cont, 0 mM NaCl + 0 mM Put; Put, 0 mM NaCl+8 mM Put; NaCl, 75 mM NaCl + 0 mM Put; NaCl+Put, 75 mM NaCl + 8 mM Put. (**a**) photosystem II Qb protein; (**b**) photosystem II protein D1; (**c**) chlorophyll a/b-binding protein CP24 precursor; (**d**) photosystem II CP43 protein; (**e**) psbA protein; (**f**) LHCII type III CAB-13; (**g**) ATP synthase beta subunit; (**h**) photosystem II CP47 protein; (**i**) photosystem II protein D2; (**j**) ATP synthase CF1 alpha subunit.

Compared with the control, 23 proteins were down-regulated, including Qb, D1, psbA, ATP synthase beta subunit, CP47, and ATP synthase alpha subunit whose abundances were decreased by 91%, 68%, 94%, 80%, 65%, and 64%, respectively (Fig. 6a,b,e,g,h,j), and six proteins, including CP24, CP43, LHCII type III, and D2 protein (Fig. 6c,d,f,i), were up-regulated by salt stress. Thus, to some extent, exogenous Put regulated salt-induced changes of thylakoid membrane proteins.

### qRT-PCR analysis of 10 candidate membrane proteins

**Figure 7:**
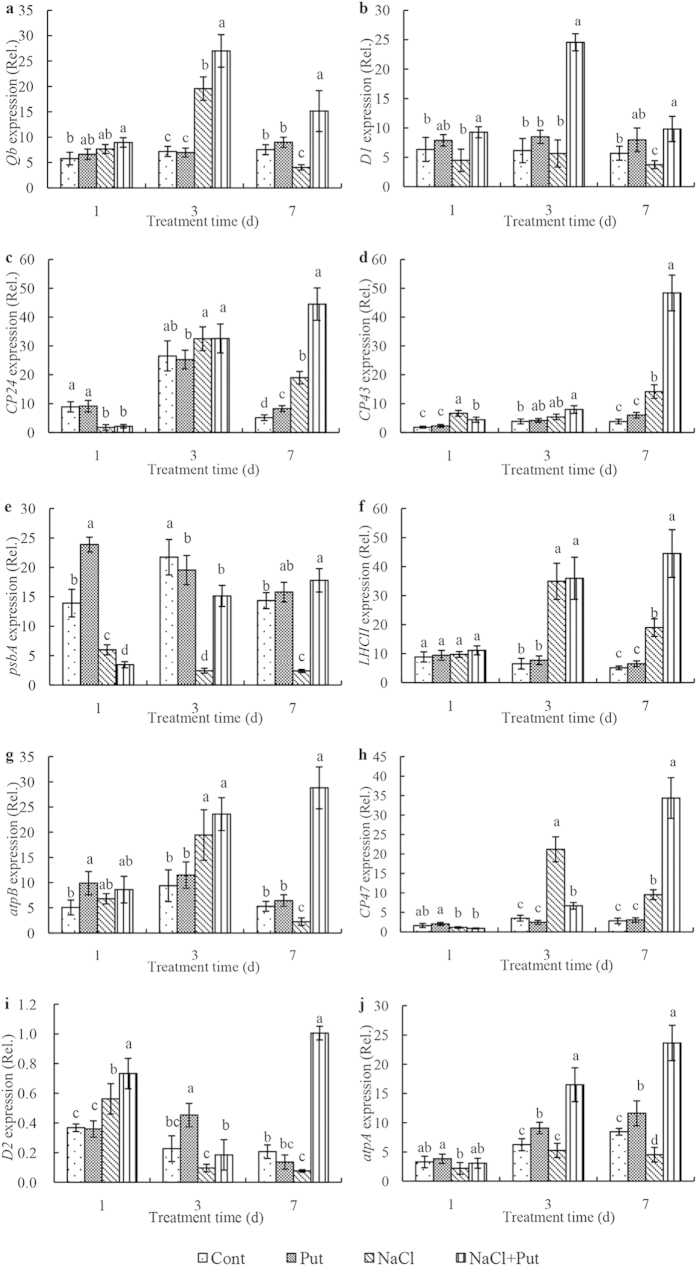
Effect of exogenous putrescine (Put) on gene expression of 10 candidate thylakoids membrane protein in leaves of cucumber plants grown in nutrient solutions with or without 75 mM NaCl for 1, 3, and 7 days. Quantitative real-time qRT-PCR was used to analysis the expression levels of the transcripts of the corresponding thylakoids membrane proteins. Each histogram represents a mean ± SE of three independent experiments. Different letters indicate significant differences between treatments (*P* < *0.05*). The data were taken on the third leaves (numbered basipetally). Cont, 0 mM NaCl + 0 mM Put; Put, 0 mM NaCl + 8 mM Put; NaCl, 75 mM NaCl + 0 mM Put; NaCl +Put, 75 mM NaCl + 8 mM Put. (**a**) *Qb*, photosystem II Qb protein; (**b**) *D1*, photosystem II protein D1; (**c**) *CP24*, chlorophyll a/b-binding protein CP24 precursor; (**d**) *CP43*, photosystem II CP43 protein; (**e**) *psbA*, psbA protein; (**f**) *LHCII*, LHCII type III CAB-13; (**g**) *atpB*, ATP synthase beta subunit; (**h**) *CP47*, photosystem II CP47 protein; (**i**) *D2*, photosystem II protein D2; (**j**) *atpA*, ATP synthase CF1 alpha subunit.

According to the results of the comparative proteomic analysis of thylakoid membranes, we selected 10 major proteins, including photosystem II Qb protein (encoded by *Qb*), photosystem II protein D1 (*D1*), chlorophyll ab-binding protein CP24 precursor (*CP24*), photosystem II CP43 protein (*CP43*), psbA (*psbA*), LHCII type III protein (*LHCII*), ATP synthase beta subunit (*atpB*), photosystem II CP47 protein (*CP47*), photosystem II protein D2 (*D2*), and ATP synthase alpha subunit (*atpA*) to analyze their transcript levels (Fig. 7a–j). As shown in Fig. 7, under non-saline conditions, exogenous Put had little effect on the relative expression of *the Qb*, *D1*, *CP24*, *CP43*, *LHCII*, *CP47*, *D2*, and *atpA* transcripts (Fig. 7a–d,f,h–j) in thylakoids, but remarkably increased the expression of *atpB* and *psbA* on day 1 (Fig. 7e). *D1* transcript abundance was decreased by NaCl stress on day 7, and *D2* expression also declined during salt stress. The expressions of the *CP47*, *CP24*, and *CP43* transcripts increased on day 7. Put significantly and continuously increased *CP47*, *CP24*, and *CP43* expression under salt stress, and also significantly increased *D1* and *D2* gene expression. *D1* expression reached its highest level on day 3, when it was 4.34-fold above that recorded under saline conditions in the absence of Put. The highest *D2* expression was observed on day 7, approximately 13.19-fold above salt-stress levels. Expressions of *LHCII* and *psbA*, which encode light-harvesting pigment protein and PSII protein complexes, were significantly altered under salt stress. On day 3, *LHCII* expression under salt stress was dramatically increased compared with the control, while *psbA* expression showed the opposite trend. Exogenous Put further increased *LHCII* and *psbA* expression under salt stress, with 2.34-fold and 7.47-fold increases observed on day 7, respectively. *Qb* expression was dramatically increased by salt stress; the highest value was recorded on day 3, when salt treatment led to 2.72-fold higher relative expression levels than in the control. Application of exogenous Put further increased *Qb* expression in salt-stressed thylakoids. Analysis of the *atpB* and *atpA* genes that encode ATPase complexes showed that *atpB* expression levels first increased, and then decreased during salt treatment, whereas *atpA* expression continuously decreased. Put increased the expression of *atpB* and *atpA* under salt stress, with up to 12.77-fold and 5.23-fold higher expression on day 7, respectively, as compared with the salt stress treatment alone. These results indicate that transcriptional levels of the studied thylakoid membrane protein-related genes varied significantly in the presence of salt stress and salt stress with Put.

### Endogenous PA content in thylakoid membranes

**Figure 8:**
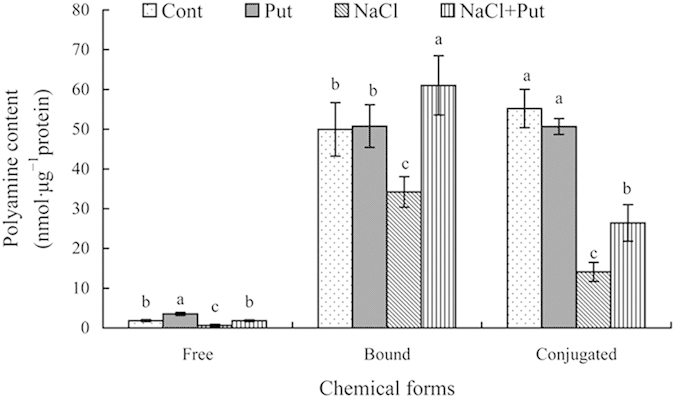
Effect of exogenous putrescine (Put) on the content of endogenous free, bound, and conjugated PAs in the thylakoid membranes of cucumber plants grown in nutrient solutions with or without 75 mM NaCl for 7 days. Each histogram represents a mean ± SE of three independent experiments. Different letters indicate significant differences between treatments (*P* < 0.05). The data were taken on the third leaves (numbered basipetally), after the final salt treatment (75 mM NaCl) for 7 days. Cont, 0 mM NaCl + 0 mM Put; Put, 0 mM NaCl + 8 mM Put; NaCl, 75 mM NaCl + 0 mM Put; NaCl+Put, 75 mM NaCl + 8 mM Put.

The differences in endogenous PA contents in thylakoid membranes among four treatments were detected using high-performance liquid chromatography. As shown in Fig. 8, three chemical forms of PAs (free, bound, and conjugated) were observed in thylakoid membranes of cucumber leaves. Compared with the control, 75 mM NaCl stressed for 7days significantly decreased the free, bound, and conjugated PA contents by 63.65%, 31.53%, and 74.49%, respectively. Exogenous Put sprayed to salt-stressed cucumber thylakoid membranes increased the free, bound, and conjugated PA contents by 177.22%, 78.41%, and 87.62%, respectively. Under non-saline conditions, except for an increase in free PAs, Put had no significant effect on the bound and conjugated PA contents in the thylakoid membranes.

## Discussion

ROS accumulation is a general feature of plants exposed to salinity stress. In this study, exogenous Put was found to suppress the accumulation of NaCl-induced H_2_O_2_ as well as Na^+^ and Cl^−^ content in chloroplasts of cucumber seedlings. This reduction is associated with reduced membrane lipid peroxidation and degradation of membrane proteins caused by NaCl, thus improving photosynthetic apparatus performance.

Excessive absorption and accumulation of Na^+^ and Cl^−^ negatively affect plant growth by decreasing photosynthetic efficiency and impairing metabolic processes^18^. PAs counteract the deleterious effects of NaCl by altering toxic ion absorption and transport^19^. The exclusion and compartmentalization of Na^+^ by both H^+^-ATPase and the Na^+^/H^+^ antiporter is a key component of plant salt tolerance^20^. Zhao and Qin^21^ have shown that exogenous application of Put or Spd partially restored the activities of V-type H^+^-ATPase, H^+^-PPase and vacuolar Na^+^/H^+^ antiporter in NaCl-stressed plants, conferring salt tolerance. In the present study, exogenous Put reduced Na^+^ levels in chloroplasts, supporting the notion that application of extracellular PAs blocks inward Na^+^ currents^22^. In addition, exogenous Put reversed the NaCl-induced down-regulation of ATPase (Spot 13, Fig. 5), thus facilitating efflux of Na^+^. Our results are in accordance with those of Janicka-Russak *et al.*^23^ who reported that PAs helped to maintain a suitable concentration of Na^+^ in the cytosol through modulation of the plasma membrane and the vacuolar H^+^-ATPase activities and thus increased cucumber salt tolerance. Moreover, similar to divalent cations such as Mg^2+^ and Ca^2+^, Put may additionally restrict Cl^−^ influx, as reported by Lorenzen *et al.*^24^. Exogenous Put may regulate Cl^−^ channels located in chloroplast envelope membranes, thereby decreasing chloroplast Cl^−^ content^25^,^26^.

In the current study, we found that plants exposed to NaCl exhibited higher H_2_O_2_ levels, with the DCF-DA-derived H_2_O_2_ signals detected mainly in guard cell chloroplasts (Fig. 2a,b). Because of the highly oxidizing metabolism and marked electron flow that occur at the PSI and PSII reaction centres of chloroplast thylakoids, chloroplasts are a major source of ROS in plant cells^27^. Higher levels of ROS in chloroplasts due to salt stress cause chloroplast dysfunction and photosynthetic damage^28^. In the present study, when the treatment time was extended, Put sprayed onto non-stressed seedlings tended to increase H_2_O_2_ levels in guard cell chloroplasts. This is probably because of the catabolization of PAs by amine oxidases in apoplasts, which can give rise to H_2_O_2_^29^. However, our study also revealed that exogenous Put effectively reduced NaCl-induced accumulation of H_2_O_2_ in chloroplasts (Fig. 2), which may be related to activation of the chloroplast antioxidant system. In a previous study, we found that exogenous application of Spm significantly increased the activities of ROS scavenging enzymes like SOD, POD, APX, and GR in chloroplasts of the salt-stressed plants^8^, and Put also had a similar effect on ROS scavenging by an antioxidant system including antioxidant compounds and antioxidant enzymes. In addition, several reports found that NO could act downstream of PAs to induce stress tolerance in plants, indeed, PAs or H_2_O_2_ induced increases in antioxidant enzymes activities is dependent on endogenous NO generation^30^,^31^.

NaCl stress induced damage to the ultrastructure of chloroplasts and thylakoids presumably as a result of increased ROS. Excessive ROS levels under salt stress lead to lipid peroxidation, which generally results in membrane destabilization^32^. This phenomenon is consistent with the disordered grana thylakoid lamellae and increased levels of osmiophilic particles on grana and stroma thylakoids observed in the present study. Unsaturated fatty acids are the major constituents of membrane lipids, and are responsible for biomembrane fluidity and stability. In this study, NaCl stress significantly increased saturated fatty acids levels, and decreased unsaturated fatty acid levels in the thylakoid membrane (Table 1), perhaps as a result of oxidation of ROS. Similar fatty acid compositional changes have been observed in plasma membranes of other plant species exposed to salt stress^33^,^34^. Many studies have demonstrated that unsaturated fatty acids are associated with plant tolerance to abiotic stresses such as cold and salinity. Desaturated fatty acids in membranes may protect the oxygen-evolving machinery from salt-induced damage and accelerate the recovery of the PSII protein complex from low-temperature photoinhibition^35^,^36^, thereby conferring stress tolerance on the photosynthetic machinery. In addition, membrane fluidity maintained by unsaturated fatty acids may affect various membrane-bound enzymes, especially the activity and synthesis of the Na^+^/H^+^ antiporter system^35^. This process may contribute to the reduced accumulation of toxic Na^+^ and thus plays an important role in plant protection against salt stress.

In this study, exogenous Put reversed NaCl-induced reduction of unsaturated fatty acid content of thylakoid membranes, increased membrane liquidity, and maintained an orderly arrangement of grana thylakoids. This outcome is consistent with the results of Mirdehghan *et al.*^37^ who found that the ability of PAs to alter the ratio of unsaturated to saturated fatty acids was responsible for the maintenance of membrane integrity and fluidity. In an experiment using vesicles prepared from soybean phospholipids, Tadolini *et al.*^38^ determined that PAs only inhibited lipid peroxidation when bound to the negative charges of the vesicle surface. On the other hand, Borrell *et al.*^39^ found that Spm inhibited the activity of lipoxygenase, which catalyzes the hydroperoxidation of unsaturated fatty acids, thus restraining lipid peroxidation and preserving thylakoid membrane integrity. Moreover, PAs act as direct free radical scavengers and can evoke the antioxidant defence system also to prevent lipid peroxidation^40^. Taken together, these findings suggest that the Put treatment in the present study counterbalanced the deleterious effects of NaCl on lipids via two processes. First, endogenous PAs may bind directly to negatively charged phospholipids of thylakoid membranes^11^, thus altering membrane fluidity and the activities of membrane-bound enzymes. Second, PAs may directly quench ROS^41^, thereby preventing lipid peroxidation.

The PA-controlled reversal of the deleterious effects of NaCl on thylakoid membrane proteins appears to be another mechanism protecting the photosynthetic apparatus from salt-induced damage. We performed BN/SDS-PAGE electrophoresis of thylakoid membrane fractions to detect major Put-induced modifications of protein structure (Fig. 6). This analysis revealed that Put may interact not only with the PSII core proteins D1, D2, and CP43, but also with the LHCII antenna complex, PSI reaction centre proteins, and the ATP synthase subunit. These results suggest that Put plays an essential role in the assembly of photosynthetic protein complexes exposed to salt stress. Several studies have shown that application of exogenous Spd regulates the expression of major PSII proteins D1 and D2 and the corresponding *D1* and *D2* gene transcripts under various environmental stresses^11^,^12^. *D1* encodes new D1 proteins and plays an important role in D1 protein turnover during stress resistance. In the present study, Put was found to alleviate salt stress-induced alteration of the transcription and translation of the D1 and D2 proteins. The protective action of Put on PSII proteins can be explained by the role of PAs in the modulation of synthesis and turnover of these proteins^7^. In a previous work, we found that Put significantly decreased the functional size of the antenna and increased reaction centre density under salt stress^1^. In the present study, Put significantly up-regulated psbA protein expression and down-regulated LHCII type III and CP24 protein expression. Put also increased the expression levels of the corresponding transcripts after 7 days of salt treatment. On a short timescale, Put has been shown to stimulate chemiosmotic ATP synthesis through regulation of the ΔpH/Δψ balance in thylakoids^42^. Over the long term, Put reduced antenna size by inducing autoproteolytic degradation of LHCII^18^. This study confirms that the transcription and translation of the LHCII supercomplex and the ATP synthase complex affected by Put plays positive roles in regulating the energy balance of thylakoid membranes and in ensuring sophisticated coordination of energy excitation and dissipation^1^,^7^,^43^. The results reported here indicate that Put most likely enhances the ability of PSII-repairing reaction centres to decrease inactivation reaction centres, thus reducing the effects of NaCl damage. Interestingly, however, the transcriptional and translational levels of CP24, CP43, CP47, and LHCII type III chlorophyll a/b-binding protein on day 7 were not entirely consistent. These differences may be a result of the different stability and update rate between the mRNA transcript and the protein^44^.

Several studies have shown that external PAs can regulate changes of endogenous PAs and reorganization of the photosynthetic apparatus in response to adverse environments^1^,^11^,^14^. Our previous study found that PAs in chloroplasts play crucial roles in protecting thylakoid membranes against the deleterious influences of salt stress^1^. In the present work, we showed that exogenous Put increased free, conjugated, and bound PA content in thylakoid membranes under salt stress (Fig. 8). Variations in PA levels in the thylakoid membranes can modify the organization of photosynthesis-related proteins. The conjugation of PAs can be catalysed by transglutaminase, which catalyses the incorporation of PAs into negatively charged thylakoid membrane proteins such as the PSII enriched submembrane fractions and LHCII. The increased endogenous PA levels in thylakoid membranes suggested that thylakoid-bound PAs exert a regulatory role in the adaptation processes of the photosynthetic apparatus^7^. Thus, these PAs may alleviate the degradation of thylakoid membrane proteins induced by salt stress, thereby stabilising of the structure and function of the photosynthetic apparatus. Interestingly, while Put sprayed onto non-stressed seedlings increased free PAs levels, this had no effect on the content of bound and conjugated PAs. The increase of free PA content may be a result of the direct absorption and transport of exogenous Put, but the increase was too weak to significantly alter the bound and conjugated PA levels.

In conclusion, this study has shown that Put can alleviate salt-induced oxidative damage and ion toxicity in the photosynthetic apparatus of cucumber seedlings. The results of the proteomic and transcript analyses of thylakoid membrane proteins suggest that Put regulates the structure and function of the photosynthetic apparatus, which may help protect the integrity of thylakoid membranes under salt stress. The possible mechanism of Put action may involve direct binding of endogenous PAs to extrinsic proteins and/or intrinsic polypeptides of PSII. This PA-protein/peptide binding, which involves electrostatic interaction related to the polycationic nature of PAs, may help enhances the photochemical efficiency of cucumber seedlings under salt stress.

## Methods

### Plant cultivation and treatment

Cucumber (*Cucumis sativus* L. cv. Jinyou No. 4) were grown in a greenhouse in trays containing quartz sand at 28 ± 1 °C day/19 ± 1 °C night, under a maximum photosynthetic photon flux density (PPFD) of about 1200 μmol m^−2^ s^−1^ with relative humidity of 75–80%. After full development of the second leaf, the seedlings were transferred into plastic containers with full-strength Hoagland solution and subjected to one of the four treatments: (a) Cont, control, plants were grown in normal Hoagland solution; (b) Put, plants were grown in Hoagland solution with 8 mM Put sprayed on leaves; (c) NaCl, salt stress treatment, plants were grown in Hoagland solution containing 75 mM NaCl; (d) NaCl+Put, plants were grown in Hoagland solution containing 75 mM NaCl, and the leaves were sprayed with 8 mM Put. For the salt stress treatment, NaCl concentrations were increased by 25 mM increments every day until a final concentration of 75 mM was reached. The containers were arranged in a completely randomized block design with three replicates per treatment (a total of 36 seedlings per treatment), comprising a total of 12 containers with 144 seedlings in the four treatments. Samples from healthy cucumber seedlings were harvested at 1, 3, and 7 days after the final concentration salt treatment, immediately frozen in liquid nitrogen and stored at −80 °C until further chemical analyses were performed.

### Isolation of intact chloroplasts and thylakoid membranes

Intact chloroplasts from the third fully expanded leaves were isolated and purified on percoll gradients according to the method of Shu *et al.*^8^. To obtain the thylakoid membranes, the intact chloroplasts were ruptured in 50 mM Hepes-KOH (pH 7.6) and 2 mM MgCl_2_ at 4 °C and the thylakoid membranes were collected by centrifugation at 14, 000 × g for 15 min^45^.

### Determination of Na^+^ and Cl^−^ contents in the chloroplasts

Boiled chloroplast extracts were used for the Na^+^ and Cl^−^ determinations^46^. Na^+^ was measured by flame-photometry (Eppendorf Netheler & Hinz, Hamburg, Germany) and Cl^−^ was determined by the AgNO_3_ titration method with 4.2% (w/v) K_2_CrO_4_ and 0.7% (w/v) K_2_Cr_2_O_7_ as mixed indicator.

### H_2_O_2_ live imaging

For H_2_O_2_ real-time production, the lower epidermis of the third leaves were torn off and incubated with 2′, 7′–dichlorofluorescein diacetate (DCF-DA) as described by Tanou *et al.*^47^ with modifications. The epidermal strips were placed in 10 mM Tris-HCl (pH 6.1) and incubated in dark for 2 h. Then the epidermal strips were transferred to 25 μM DCF-DA dissolved in Tris-HCl and incubated for 20 min. After washing twice with Tris-HCl buffer for 30 min, images were visualized using a Leica TCS-SP confocal laser scanning microscope (CLSM; Leica DM RXA; Lecia; Gemany). The intensities of the DCF-DA fluorescence were analysed using Image Leica SP2 software and expressed as relative value.

### Fatty acid composition of thylakoid membranes

Thylakoid membrane lipids were separated from the isolated thylakoid membranes and the fatty acids were analysed according to the method of Zhang *et al.*^48^. The lipids extracts were separated by gas chromatography (HP6890, Agilent). An Agilent HP-INNOWax column (33 m × 0.25 mm) was packed with polyethylene glycol. Hydrogen flame ionization was detected at 230 °C, and the column temperature was programmed to rise from 170 °C to 210 °C at 5 °C per min. Chromatograms were recorded and peak areas were calculated to measure the fatty acids levels. Peaks were identified by comparisons against several external qualitative standards.

### Observation of the ultrastructure of the chloroplasts

After 7 days of 75 mM NaCl treatment, the third leaves (numbered basipetally) were collected and the ultrastructure of the chloroplasts was observed as described by Shu *et al.*^1^. Leaf segments were cut into pieces of approximately 1 mm^2^ immersed in a mixture of 3% glutaraldehyde and 1% formaldehyde in a 0.1 M phosphate buffer (pH 7.4) for 2 h (primary fixation), then in 2% osmic acid in the same buffer for 2 h (secondary fixation). After dehydration in acetone and embedding in Durcupan ACM, ultra-thin sections of the leaf pieces were cut, stained with uranium acetate and lead citrate in series and examined under a HITACHI transmission electron microscope (Carl Zeiss, Göttingen, Germany) at an accelerating voltage of 80 kV.

### Solubilization of thylakoid membrane proteins

Thylakoid membranes were resuspended in solubilisation buffer containing 25 mM BisTris-HCl (pH 7.0), 20% glycerol and 2% *n*-dodecyl-β-D-maltoside (Sigma). After incubation for 30 min on ice, samples were centrifuged at 14, 000 × g for 3 min to remove insoluble material. The supernatant was supplemented with a certain volume of sample buffer [1% (w/v) Coomassie brilliant blue G-250, 0.1 M BisTris-HCl, pH 7.0, 30% sucrose and 0.5 M 6-amino-n-caproic acid]. Dye-labelled protein samples were directly loaded onto Blue-native gels.

### Two-dimensional Blue-native/SDS-polyacrylamide gel electrophoresis

Two-dimensional Blue-native/SDS-polyacrylamide gel electrophoresis (2D BN/SDS-PAGE) was carried out as described by Reisinger & Eichacker^49^ with minor modifications. The first dimension, BN-PAGE, is a native polyacrylamide gel in which a gradient gel of 5%–13.5% acrylamide was used. The anode buffer contained 50 mM BisTris-HCl, pH 7.0 and the cathode buffer contained 50 mM Tricine, 15 mM BisTris and 0.01% (w/v) Coomassie brilliant blue G-250. The gel was run at 4 °C. When the BN-PAGE was completed, the gel was equilibrated in 6 M urea, 5% (w/v) SDS, 10% β-mercaptoethanol, 20% (v/v) glycerol, and 50 mM Tris-HCl (pH 7.0) for 20 min. After washing with deionized water for three times, individual lanes were cut and inserted into the gel loading hole of the second dimension, SDS-PAGE, which was carried out as described by Laemmli^50^. Protein spots were visualized using Coomassie brilliant blue R-250.

### Image acquisition and data analysis

The stained gels were scanned using the Image scanner III (GE Healthcare). The images were analyzed with Imagemaster^TM^ 2D Platinum software version 6.0 (GE Healthcare). Three gels for each treatment from three independent experiments were used for the analysis. The intensities of spots were quantified based on the ratio of the volume of a single spot to the whole set of spots. Only spots with quantitative changes of at least 1.5-fold in abundance that were reproducible in three replicates were used for mass spectrometry.

### MALDI-TOF/TOF MS analysis and database searching

Differentially expressed protein spots were extracted, processed, and analyzed using an ABI 4800 Proteomics Analyzer MALDI-TOF/TOF MS (Applied Biosystems, Foster City, CA) as described previously by Li *et al.*^51^. Internal mass calibration was performed using trypsin autodigestion products (842.51 kDa and 2211.10 kDa). The resulting peptide mass lists were used to search the NCBI database (http://www.ncbi.nlm.nih.gov/), with the MOWSE search parameter criteria as follows: trypsin specificity, one missed cleavage site, cysteine carbamidomethylation, acrylamide modified cysteine, methionine oxidation, similarity of pI, relative molecular mass specified, and minimum sequence coverage of 15%. All identified proteins had a MASCOT score greater than a significance level of *P* < 0.05. The identification of proteins was repeated at least once using spots from different gels and the classification of identified proteins was based on gene ontology biological process and molecular functions terms annotated using the UniProt Knowledgebase (UniProtKB, http://www.uniprot.org/).

### RNA isolation, cDNA synthesis, and quantitative real-time (qRT-PCR) analysis

Total RNA was isolated from cucumber leaves using Trizol reagent (Takara, Otsu, Japan), as described in the manufacturer’s protocol. For all samples, 1 μg of total RNA was reverse transcribed into cDNA using a SuperScript First-strand Synthesis System for qRT-PCR, according to the manufacturer’s instructions. The genes used for the qRT-PCR analysis were selected from the peptide sequences obtained after mass analysis. Primers were designed according to NCBI and Cucumber Genome Database (cucumber.genomics.org.cn) (Table 3). qRT-PCR was performed on a StepOnePlus™ Real-Time PCR System (Applied Biosystems) using a SYBR^®^ Premix Ex Taq™ II (Tli RNaseH Plus) kit (Takara). The PCR reactions were carried out in a 20 μL reaction mixture containing 2 μL cDNA (diluted 1:5), 10 μL SYBR^®^ Premix Ex Taq™ II (2×), 0.8 μL 10 μM of each specific primer (Table 3), 0.4 μL ROX reference dye, and 6 μL ddH_2_O. Reactions were carried out in triplicate and the thermocycler conditions were: 95 °C for 30 s, followed by 40 cycles of 95 °C for 5 s, 60 °C for 30 s, and a final extension of 30 s at 60 °C. Relative expression was calculated using the 2^−ΔΔCt^ method, where the relative mRNA expression level was normalized against *actin* (the internal standard gene) and compared with the control.

### Analysis of endogenous polyamines in the thylakoid membranes

The contents of free, bound, and conjugated forms of endogenous PAs in the thylakoid membranes were analyzed according to a method described by Shu *et al.*^1^. An aliquot of thylakoid membranes, prepared as described above, was incubated in 1.6 mL of 5% (w/v) cold perchloric acid (PCA) for 1 h on ice. After centrifugation for 20 min at 12,000 × g, the supernatant was used to determine free and conjugated PAs and the pellet was used to determine bound PAs. PAs were assayed using a high-performance liquid chromatography 1200 series system (Agilent Technologies, Santa Clara, CA) with a C18 reverse phase column (4.6 mm by 250 mm, 5 μm Kromasil) and a two solvent system including a methanol gradient (36%–64%, v/v) at a flow rate of 0.8 mL·min^–1^.

### Statistical analysis

All data were statistically analyzed with SAS 13.0 software (SAS Institute, Inc., Cary, NC, USA) using Duncan’s multiple range test at a 0.05 level of significance.

## Additional Information

**How to cite this article**: Shu, S. *et al.* The role of putrescine in the regulation of proteins and fatty acids of thylakoid membranes under salt stress. *Sci. Rep.* **5**, 14390; doi: 10.1038/srep14390 (2015).

## Figures and Tables

**Table 1:** Effect of exogenous putrescine on the fatty acid composition of thylakoid membranes in salt-stressed cucumber plants.

**Fatty acid Composition (mg·mg^−1^Chl)**	**Treatments**			
	**Cont**	**Put**	**NaCl**	**NaCl+Put**
Tetradecanoic acid	0.708 ± 0.219 b	0.739 ± 0.038 b	4.742 ± 0.576 a	1.859 ± 0.464 b
Palmitic acid	7.223 ± 1.072 c	6.431 ± 0.339 c	17.526 ± 0.205 a	13.214 ± 0.898 b
Stearic acid	6.015 ± 0.427 b	8.450 ± 0.352 a	3.993 ± 0.570 c	5.289 ± 0.468 bc
Palmitoleic acid	5.888 ± 1.267 ab	7.279 ± 0.971 a	1.656 ± 0.259 c	3.282 ± 0.217 bc
Hexadecatrienoic acid	4.802 ± 0.269 a	4.578 ± 0.179 a	1.988 ± 0.499 b	4.685 ± 0.462 a
Oleic acid	11.580 ± 0.446 a	8.930 ± 0.407 b	4.486 ± 0.472 c	5.253 ± 0.302 c
Linoleic acid	10.045 ± 0.279 a	9.329 ± 0.894 a	4.209 ± 0.775 b	11.542 ± 1.175 a
Linolenic acid	15.798 ± 0.484 a	15.819 ± 0.496 a	2.604 ± 0.389 c	12.958 ± 0.642 b
Saturated fatty acid (SFA)	13.238 ± 0.660 c	14.882 ± 0.658 c	21.519 ± 0.672 a	18.503 ± 1.314 b
Unsaturated fatty acid (UFA)	48.113 ± 0.898 a	45.934 ± 0.767 a	14.943 ± 1.405 c	37.720 ± 1.647 b
UFA/SFA	3.648 ± 0.136 a	3.095 ± 0.103 a	0.694 ± 0.115 c	2.071 ± 0.236 b
Total fatty acid	62.059 ± 1.253 a	61.554 ± 1.370 a	41.204 ± 1.439 b	58.082 ± 0.697 a

*Note:* Each value is the mean ± SE of three independent experiments. Different letters after the values indicate significant differences between treatments (*P* < *0.05*). The data were taken on the third leaves, numbered basipetally, after the final salt treatment (75 mM NaCl) for 7 days. Cont, 0 mM NaCl + 0 mM Put; Put, 0 mM NaCl + 8 mM Put; NaCl, 75 mM NaCl + 0 mM Put; NaCl+Put, 75 mM NaCl + 8 mM Put.

**Table 2:** Differentially synthesised proteins identified by MALDI-TOF-MS and LC–ESI-MS/MS from the spots in BN/SDS-PAGE in Fig. 5.

**Spots**	**Protein name**	**Source**	**Accession No**	**Theoretical Mr/PI**	**Score**
1	ATP synthase CF1 alpha subunit	*Atropa belladonna*	gi|28261702	55476.1/5.26	355
2	ATP synthase beta subunit	*Cucumis sativus*	gi|14718022	52002.1/5.13	655
3	ATP synthase beta subunit	*Cucumis sativus*	gi|14718022	520021/5.13	640
4	putative magnesium subunit chlD	*Oryza sativa* Japonica Group	gi|32129323	81025.0/5.38	47
5	LHCII type I chlorophyll a/b-binding protein	*Vigna radiate*	gi|9587207	27950.1/5.13	114
6	LHCII type I chlorophyll a/b-binding protein	*Vigna radiate*	gi|9587205	28003.1/5.14	149
7	chlorophyll a/b-binding protein CP26 in PS II	*Brassica juncea*	gi|1644289	30572.9/6.00	78
8	putative elicitor-inducible cytochrome P450	*Oryza sativa* Japonica	gi|51091420	59812.2/8.80	52
9	ATP synthase CF1 alpha subunit	*Atropa belladonna*	gi|28261702	55476.1/5.26	409
10	ATP synthase beta subunit	*Cucumis sativus*	gi|14718022	52002.1/5.13	739
11	F-ATPase subunit alpha	*Nicotiana tabacum*	gi|55977763	55477.1/5.14	298
12	H(+)-transporting ATP synthase	*Dais cotinifolia*	gi|4995109	52200.1/5.15	310
13	ATPase subunit I	*Spinacia oleracea*	gi|7636090	47260.7/5.47	210
14	ATP synthase beta subunit	*Cucurbita pepo*	gi|14718024	52043.0/5.04	167
15	photosystem II CP47 protein	*Gunnera chilensis*	gi|27446459	53482.0/6.07	294
16	photosystem II CP47 protein	*Roystonea princep*	gi|37721438	54951.7/6.02	188
17	photosystem II P680 chlorophyll A apoprotein	*Oenothera argillicola*	gi|16914273	56076.5/6.40	347
18	photosystem II Qb protein	*Bruguiera gymnorhiza*	gi|8131597	14347.0/4.71	170
19	psbA	*Conocephalum conicum*	gi|5689006	38866.4/5.30	79
20	photosystem II CP43 protein	*Chloranthus* japonicas	gi|27435869	47413.3/6.64	331
21	photosystem II CP47 protein	*Dasypogon hookeri*	gi|37721411	55007.8/6.13	480
22	photosystem II protein D1	*Lotus japonicus*	gi|13518418	39064.5/5.11	167
23	chlorophyll a/b-binding protein precursor	*Citrus limon*	gi|28630973	22937.5/5.25	98
24	photosystem II CP47 protein	*Gunnera chilensis*	gi|27446459	53482.0/6.07	244
25	ATP synthase beta subunit	*Austrobaileya scandens*	gi|7687960	53571.8/5.15	48
26	chlorophyll a/b-binding protein CP24 precursor	*Vigna radiata*	gi|8954298	27344.1/6.15	186
27	LHCII type III CAB-13	*Solanum lycopersicum*	gi|115794	28661.4/5.09	80
28	Methyltransferase	*Arabidopsis thaliana*	gi|15232158	31799.4/8.51	63
29	ATP synthase epsilon subunit	*Nasa triphylla*	gi|22796597	14527.8/5.43	148
30	photosystem II D2 protein	*Columnea sp. Lindqvist and Albert*	gi|22858666	14299.2/5.07	290
31	ATPase subunit I	*Spinacia oleracea*	gi|7636090	47260.7/5.26	301
32	photosystem I P700 chlorophyll a apoprotein A2	*Nicotiana tabacum*	gi|11465954	82470.8/6.72	89

*Note:* A MASCOT search against the NCBInr database revealed the identity of 29 of the 32 proteins. The remaining three proteins (spots 4, 8, and 25, Fig. 5) had matching scores significantly lower than the threshold and may represent unknown proteins. Theoretical molecular weight (Mr) and pI values were calculated using the ProtParam tool available at http://us.expasy.org/.

**Table 3:** Primers used for qRT-PCR analysis and their target genes.

**Gene**	**Product length (*bp*)**	**Annealing temp. (°C)**	**Primer sequence**
*D1*	167	60.1	S 5′-TCTGCAGCTATCGGTTTGC-3′
		59.7	As 5′-GGACGCATACCCAGACAGA-3′
*atpB*	160	60.0	S 5′-GCGGGCCTATTCCATCTT-3′
		60.2	As 5′-TCGGGGTAGTTGCTGGAA-3′
*atpA*	179	60.0	S 5′-TGATGCCTGTCGCTGTGT-3′
		60.0	As 5′-AGTCGCCTCAAACGCAAC-3′
*D2*	186	60.0	S 5′-TACCCTTCCTGCCATCCA-3′
		60.1	As 5′-TGCTCAAACGCTTGGTGA-3′
*LHCII*	210	55.8	S 5′-AGCCACCATCCTACCTTACC-3′
		56.3	As 5′-CAGCCTTGAACCAAACAGC-3′
*CP43*	182	58.6	S 5′-GAATACGGTAAAGTGGCTCCTG-3′
		50.0	As 5′-CCTAAAGCACCTAAACTATAAGAC-3′
*CP47*	298	49.5	S 5′-GGTCCTGGAATATGGGTA-3′
		50.0	As 5′-TTGCTGGAACTATGTGGTA-3′
*Qb*	148	48.8	S 5′-AAACTCTAATACAGGCATACG-3′
		38.1	As 5′-AAATCGGACACTTTTCTCA-3′
*CP24*	252	52.8	S 5′-CTGGTGTTAGAGGTGGTGG-3′
		57.9	As 5′-CGAAGGAGAATGGAGCAA-3′
*psbA*	249	48.5	S 5′-CGGTGGTCCTTATGAGAT-3′
		50.0	As 5′-TTACGGTCGTTCCTTATTC-3′
*actin*	290	58.0	S 5′-CCGTTCTGTCCCTCTACGCTAGTG-3′
			As 5′-GGAACTGCTCTTTGCAGTCTCGAG-3′

*Note: D1*, photosystem II protein D1; *atpB*, ATP synthase beta subunit; *atpA*, ATP synthase CF1 alpha subunit; *D2*, photosystem II protein D2; *LHCII*, LHCII type III chlorophyll a/b-binding protein; *CP43*, photosystem II CP43 protein; *CP47*, photosystem II CP47 protein; *Qb*, photosystem II Qb protein; *CP24*, chlorophyll ab-binding protein CP24 precursor; *psbA*, psbA protein.
